# Supplementary material for: Neural dissociation of attention and working memory through inhibitory control
Source: Nat Commun. 2025 Dec 2;17:22. doi: 10.1038/s41467-025-66553-7 (PMC12764954; doi:10.1038/s41467-025-66553-7)
Supplement: Supplementary file 1 — Supplementary Information [file 41467_2025_66553_MOESM1_ESM.pdf]

Neural Dissociation of Attention and Working Memory through Inhibitory Control

Supplementary Information

Supplementary Table 1. MNI coordinates of regions identified in the statistical activation maps.

Region	Max T	Voxel	Peak MNI Coordinates		
			x	y	z
Attribute Amnesia task					
PRE > POST					
SMG.R	4.99	68	55	-44	37
POST > PRE					
dlPFC.L	4.47	121	-44	25	22
SMA.L	4.70	93	-2	13	58
vmPFC.L	4.28	62	-5	25	-20
Face-localizer task					
ATL.L	5.14	65	-32	-11	-38
ATL.R	3.53	93	31	-2	-50
FFA.L	6.27	41	-47	-56	-23
FFA.R	5.71	110	46	-59	-20

Note: Regions were defined from group-level statistical maps thresholded at $p < 0.01$ (FDR-corrected at the voxel level), with a minimum cluster extent of >40 voxels. Coordinates are reported in LPI (Left-Posterior-Inferior) orientation. Abbreviations: SMG.R, right supramarginal gyrus; dlPFC.L, left dorsolateral prefrontal cortex; SMA.L, left supplementary motor area; vmPFC.L, left ventromedial prefrontal cortex; ATL.L, left anterior temporal lobe; FFA.R, right fusiform face area. n = 49 participants.

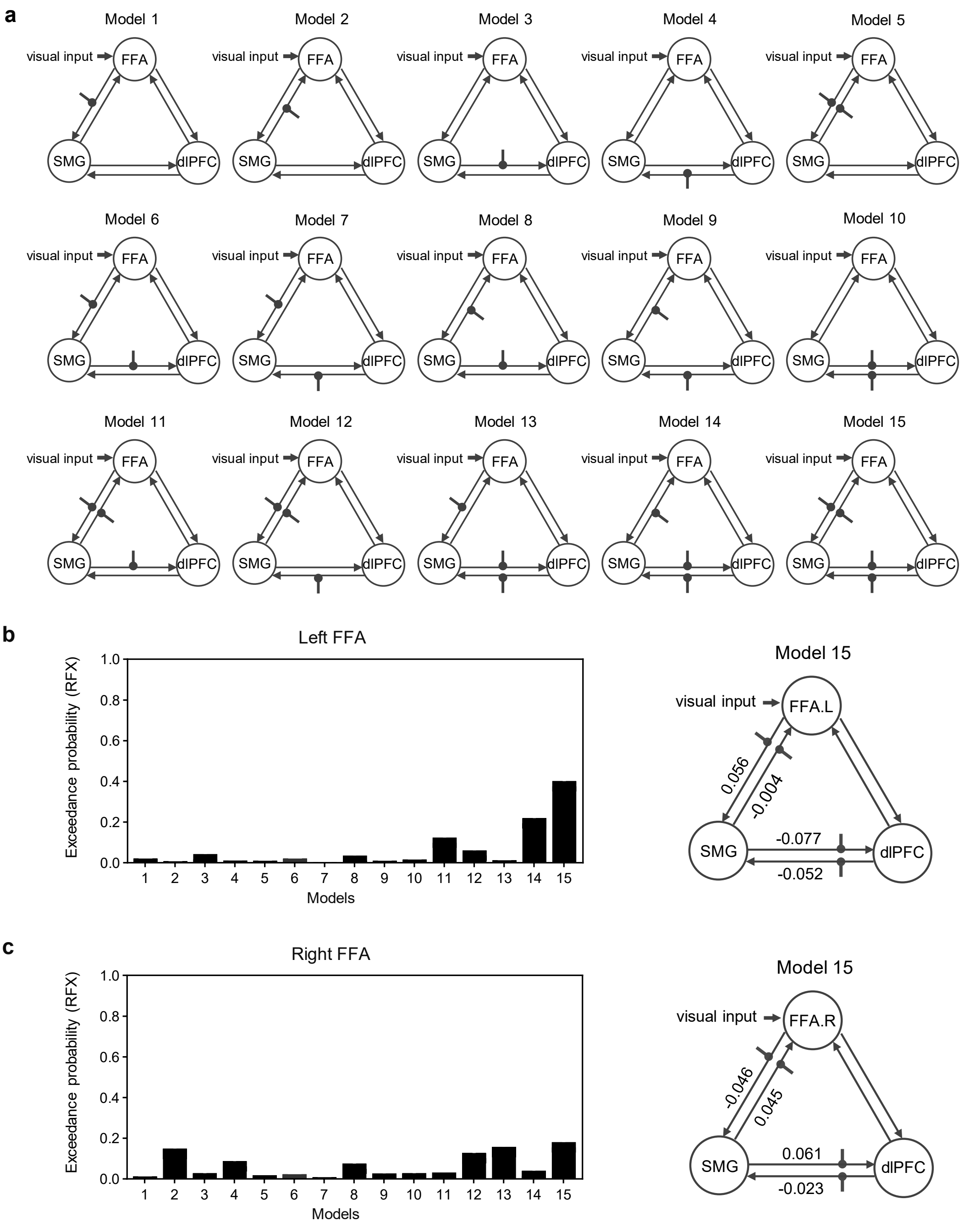

Supplementary Fig. 1. DCM results for inhibitory circuits regulating face category representation. (**a**) Model space (15 variants) with dotted arrows indicating modulatory influences in the FFA/SMG/dlPFC network. (**b**) Network with the left FFA (Left: exceedance probabilities; Right: modulatory parameters of the winning model; values reflect group-mean parameters across participants, n = 49). (**c**) As in panel (b), for the right FFA. Across participants, one-sample two-sided t-tests on modulatory parameters in both winning models showed no significant effects (uncorrected for multiple comparisons). Source data are provided as a Source Data file.

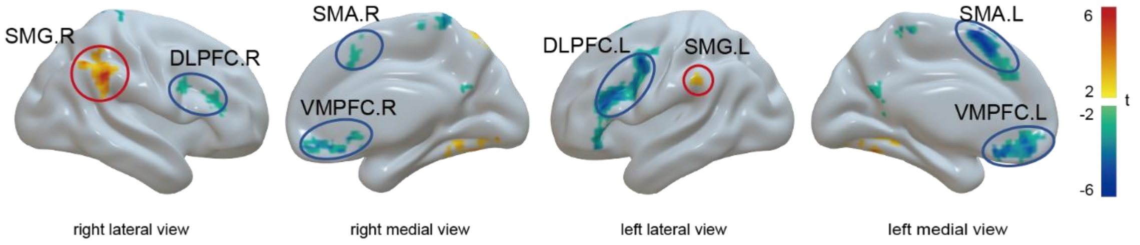

Supplementary Fig. 2. Whole-brain activation results for the attribute amnesia task using a more lenient statistical threshold. Broader activation patterns are visualized using an exploratory group-level GLM analysis of the contrast "pre-surprise > post-surprise" at a more lenient threshold ($p < 0.005$, uncorrected; cluster extent > 40 voxels). Warm colors (red/yellow) indicate greater activation in the pre-surprise condition, while cool colors (blue/green) indicate greater activation in the post-surprise condition. Notable clusters were observed in bilateral SMG (supramarginal gyrus), dlPFC (dorsolateral prefrontal cortex), SMA (supplementary motor area), and vmPFC (ventromedial prefrontal cortex). n = 49 participants.

Supplementary Table 2. MNI coordinates of regions identified under a more lenient threshold for later bilateral DCM analysis.

Region	Max T	Voxel	Peak MNI Coordinates		
			x	y	z
Attribute Amnesia task					
PRE > POST					
SMG.L	3.64	75	-68	-35	34
POST > PRE					
dlPFC.R	3.55	44	49	34	19

Note: Additional contralateral homologous regions were localized in the attribute amnesia experiment, based on group-level statistical maps thresholded at $p < 0.005$ (uncorrected) with a cluster extent >40 voxels (see Supplementary Fig. 2). In addition to the right SMG and left dlPFC reported in Supplementary Table 1, this analysis identified activation peaks in the left SMG and right dlPFC, enabling bilateral DCM modeling. Additionally, separate coordinates for the right SMA and right vmPFC were not reported, as the clusters in

these regions were bilateral and spatially contiguous, precluding reliable hemisphere-specific localization. Coordinates are reported in LPI orientation. n = 49 participants.

Extended DCM Analysis Using Full-Modulation Model

Supplementary Methods. In addition to the hypothesis-driven DCM analysis reported in the main text, we conducted a complementary data-driven analysis using a fully connected, full-modulation model to explore condition-specific modulatory interactions among six bilateral regions of interest (ROIs): the left and right SMG, ATL, and dlPFC, including all inter-regional and self-connections.

ROIs were defined based on AFNI-derived peak coordinates from the main group-level contrasts (Supplementary Table 1). Contralateral homologous regions that did not show significant activation in the main analysis were identified using a more lenient statistical threshold ($p < 0.005$, uncorrected; Supplementary Table 2). Following standard DCM procedures for data-driven analysis[1,2], we used SPM's volume of interest (VOI) module to extract the principal eigenvariate time series from each ROI. For each participant, subject-specific local maxima were identified within spherical search volumes (8 mm outer radius, 6 mm inner radius) centered on group-level peak coordinates, using an uncorrected threshold of $p < 0.05$[1]. Valid eigenvariates across all six ROIs were obtained for only 22 of 49 participants. Most exclusions arose from unsuccessful extraction in the bilateral ATLs, which lie near the ear canal and are susceptible to magnetic field inhomogeneities[3,4]. Similar challenges have been reported in fMRI studies of ATL face selectivity[4–6] and in previous DCM work[1,7]. The extended data-driven DCM analysis was therefore conducted on these 22 participants.

The driving inputs reflected the visual stimuli presented during both the pre- and post-surprise conditions and were modeled as targeting the bilateral ATL. The intrinsic connectivity matrix included all possible connections among the six ROIs, including inter-regional and self-connections. The modulatory matrix captured condition-specific effects of the pre-surprise condition, allowing modulation on all intrinsic connections. Group-level

model estimation was performed using Parametric Empirical Bayes (PEB), and Bayesian Model Averaging (BMA) was applied to compute posterior probabilities for each connection[2].

Supplementary Results. The data-driven analysis using PEB-BMA confirmed and extended our main findings. As shown in Supplementary Fig. 3, among all conditionally modulated connections, the strongest evidence for negative modulation (posterior probability > 95%; posterior expectation = -0.699) was observed for the connection from the right SMG to the left ATL. In contrast, no significant modulatory effects were observed from the SMG to the dlPFC, reinforcing the specificity of SMG-ATL interactions over SMG-dlPFC pathways. These results are consistent with the findings reported in the main text and provide direct support for our core hypothesis that the SMG exerts increased inhibitory control over perceptual representations in the ATL to regulate access to working memory.

While additional significant modulatory effects were identified—for example, between bilateral homologous regions (e.g., left ATL-right ATL, left SMG-right SMG, left dlPFC-right dlPFC)—these connections were not central to our theoretical framework and are therefore not discussed further (see Supplementary Table 3 for complete results).

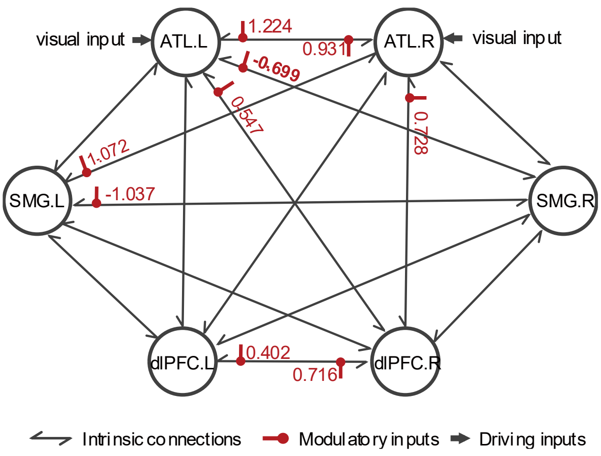

Supplementary Fig. 3. Full-modulation model results from the extended DCM analysis. Six bilateral ROIs were included: left and right SMG, ATL, and dlPFC. Each region was bidirectionally connected to the others (double-headed arrows) and included a self-connection (not shown for clarity). Visual inputs from both pre- and post-surprise conditions entered the bilateral ATL, and the pre-surprise condition modulated all inter-regional and self-connections. Significant modulatory effects (posterior probability > 95%) are

indicated by red arrows. Notably, the analysis revealed strong inhibitory modulation from the right SMG to the left ATL (modulatory effect = -0.699), suggesting enhanced top-down suppression of perceptual representations in ATL. No significant modulatory effects were observed from SMG to dlPFC, reinforcing the specificity of SMG-ATL interactions. Other significant connections (e.g., between bilateral homologues) were observed but are not central to our theoretical model. n = 22 participants.

Supplementary Table 3. Parameter estimates from the full-modulation model.

Connection	Modulatory effect	Posterior probability
RDLPFC → RDLPFC	-0.307	0.518
RDLPFC → RSMG	0.198	0.663
**RDLPFC → RATL**	**0.728**	**1.000**
**RDLPFC → LDLPFC**	**0.402**	**1.000**
RDLPFC → LSMG	0.000	0.000
**RDLPFC → LATL**	**0.547**	**1.000**
RSMG → RDLPFC	0.000	0.000
**RSMG → RSMG**	**-1.650**	**1.000**
RSMG → RATL	0.000	0.000
RSMG → LDLPFC	-0.475	0.830
**RSMG → LSMG**	**-1.037**	**1.000**
**RSMG → LATL**	**-0.699**	**1.000**
RATL → RDLPFC	0.000	0.000
RATL → RSMG	0.000	0.000
**RATL → RATL**	**-2.102**	**1.000**
RATL → LDLPFC	0.000	0.000
**RATL → LSMG**	**1.072**	**1.000**
**RATL → LATL**	**1.224**	**1.000**
**LDLPFC → RDLPFC**	**0.716**	**1.000**
LDLPFC → RSMG	0.000	0.000
LDLPFC → RATL	0.000	0.000
**LDLPFC → LDLPFC**	**-1.077**	**1.000**
LDLPFC → LSMG	0.000	0.000
LDLPFC → LATL	0.000	0.000
LSMG → RDLPFC	0.000	0.000
LSMG → RSMG	0.308	0.673
LSMG → RATL	0.203	0.507
LSMG → LDLPFC	0.000	0.000
LSMG → LSMG	-0.803	0.848
LSMG → LATL	0.000	0.000
LATL → RDLPFC	-0.588	0.949
LATL → RSMG	0.000	0.000
**LATL → RATL**	**0.931**	**1.000**
LATL → LDLPFC	0.000	0.000
LATL → LSMG	0.539	0.853
**LATL → LATL**	**-2.187**	**1.000**

Note: Estimated modulatory effects under the pre-surprise condition for all bidirectional and self-connections among six ROIs in the full-modulation DCM model: bilateral ATL, SMG, and dlPFC (L = left, R = right). The strength and direction of modulatory effects are indexed by posterior expectations (Ep); connections with posterior probability (Pp) > 95% are highlighted in bold. n = 22 participants.

Supplementary References

1. Zeidman, P. *et al.* A guide to group effective connectivity analysis, part 1: First level analysis with DCM for fMRI. *NeuroImage* **200**, 174–190 (2019).

2. Zeidman, P. *et al.* A guide to group effective connectivity analysis, part 2: Second level analysis with PEB. *NeuroImage* **200**, 12–25 (2019).

3. Collins, J. A. & Olson, I. R. Beyond the FFA: The role of the ventral anterior temporal lobes in face processing. *Neuropsychologia* **61**, 65–79 (2014).

4. Rajimehr, R., Young, J. C. & Tootell, R. B. H. An anterior temporal face patch in human cortex, predicted by macaque maps. *Proc. Natl. Acad. Sci.* **106**, 1995–2000 (2009).

5. Tsao, D. Y., Moeller, S. & Freiwald, W. A. Comparing face patch systems in macaques and humans. *Proc. Natl. Acad. Sci.* **105**, 19514–19519 (2008).

6. Pinsk, M. A. *et al.* Neural representations of faces and body parts in macaque and human cortex: A comparative fMRI study. *J. Neurophysiol.* **101**, 2581–2600 (2009).

7. Jamieson, A. J. *et al.* A brain model of altered self-appraisal in social anxiety disorder. *Transl. Psychiatry* **13**, 344 (2023).
