# Supplementary material for: Neural dissociation of attention and working memory through inhibitory control
Source: Nat Commun. 2025 Dec 2;17:22. doi: 10.1038/s41467-025-66553-7 (PMC12764954; doi:10.1038/s41467-025-66553-7)
Supplement: Supplementary file 2 — Reporting Summary [file 41467_2025_66553_MOESM2_ESM.pdf]

nature portfolio

Corresponding author(s): Hui Chen,Pengmin Qin

Last updated by author(s): Oct 16, 2025

Reporting Summary

Nature Portfolio wishes to improve the reproducibility of the work that we publish. This form provides structure for consistency and transparency in reporting. For further information on Nature Portfolio policies, see our Editorial Policies and the Editorial Policy Checklist.

Statistics

For all statistical analyses, confirm that the following items are present in the figure legend, table legend, main text, or Methods section.

n/a	Confirmed	
☐	☒	The exact sample size (*n*) for each experimental group/condition, given as a discrete number and unit of measurement
☐	☒	A statement on whether measurements were taken from distinct samples or whether the same sample was measured repeatedly
☐	☒	The statistical test(s) used AND whether they are one- or two-sided<br>*Only common tests should be described solely by name; describe more complex techniques in the Methods section.*
☐	☒	A description of all covariates tested
☐	☒	A description of any assumptions or corrections, such as tests of normality and adjustment for multiple comparisons
☐	☒	A full description of the statistical parameters including central tendency (e.g. means) or other basic estimates (e.g. regression coefficient) AND variation (e.g. standard deviation) or associated estimates of uncertainty (e.g. confidence intervals)
☐	☒	For null hypothesis testing, the test statistic (e.g. *F*, *t*, *r*) with confidence intervals, effect sizes, degrees of freedom and *P* value noted<br>*Give P values as exact values whenever suitable.*
☐	☒	For Bayesian analysis, information on the choice of priors and Markov chain Monte Carlo settings
☒	☐	For hierarchical and complex designs, identification of the appropriate level for tests and full reporting of outcomes
☐	☒	Estimates of effect sizes (e.g. Cohen's *d*, Pearson's *r*), indicating how they were calculated

Our web collection on statistics for biologists contains articles on many of the points above.

Software and code

Policy information about availability of computer code

Data collection	Behaviour data were collected using MATLAB2018b with Psychtoolbox-3. MRI data were acquired using a Siemens 3T Magnetom Prisma Fit scanner. tDCS using a Soterix Medical (New York,USA)device and HD-explore version 6.0. TMS using Visor 2.0 neuronavigation software (ANT Neuro, Netherlands), tracked with a Polaris Spectra motion tracking system (NDI, Canada), and magnetic stimulation was delivered via a figure-of-eight coil (outer diameter: 70 mm) connected to an NS5000 stimulator (YIRUIDE, Wuhan, China).
Data analysis	fMRI data analysis were performed using AFNI software package(version 21.2.03) and SPM12. Behaviour data,tDCS data and TMS data analysis were conducted using MATLAB 2018b and PYTHON 3.8. Example scripts are available on OSF (DOI: 10.17605/OSF.IO/V2QN9). No additional custom code beyond official package tutorials was developed.

For manuscripts utilizing custom algorithms or software that are central to the research but not yet described in published literature, software must be made available to editors and reviewers. We strongly encourage code deposition in a community repository (e.g. GitHub). See the Nature Portfolio guidelines for submitting code & software for further information.

Data

Policy information about availability of data

All manuscripts must include a data availability statement. This statement should provide the following information, where applicable:

- Accession codes, unique identifiers, or web links for publicly available datasets
- A description of any restrictions on data availability
- For clinical datasets or third party data, please ensure that the statement adheres to our policy

The tDCS/TMS data and derived fMRI data together with the exact numerical values underlying all main and supplementary figures/tables, are available on OSF (DOI: [https://doi.org/10.17605/OSF.IO/V2QN9). Source data are provided with this paper.

Research involving human participants, their data, or biological material

Policy information about studies with human participants or human data. See also policy information about sex, gender (identity/presentation), and sexual orientation and race, ethnicity and racism.

Reporting on sex and gender	We collected the sex information for each participant through self-reporting of their sex (binary choice: male or female) prior to the commencement of the experiment. Sex was not considered as a factor in this study design because we tested the general cognitive ability and no prior studies have reported the sex effect on attribute amnesia paradigm and change detection paradigm.
Reporting on race, ethnicity, or other socially relevant groupings	All participants were Chinese adults. The study complied with ethical regulations for human research. This single-country sample may limit cross-cultural generalizability; culture was not an a priori factor and the study was not powered for culture-based analyses.
Population characteristics	Participants were right-handed young adults (aged 18–35 years) with normal or corrected-to-normal vision and no history of face-recognition deficits, color-vision deficits, or neurological or psychiatric disorders.
Recruitment	All participants were recruited from universities in Guangzhou, China, including both undergraduate and graduate students. They had normal or corrected-to-normal visual acuity, no deficits in facial recognition or color vision, and no prior experience with similar experiments. All participants provided informed consent and received monetary compensation for their time.
Ethics oversight	The study was approved by the Research Ethics Board of the School of Psychology at South China Normal University. All participants signed informed consent forms prior to participation in the study.

Note that full information on the approval of the study protocol must also be provided in the manuscript.

Field-specific reporting

Please select the one below that is the best fit for your research. If you are not sure, read the appropriate sections before making your selection.

☒ Life sciences ☐ Behavioural & social sciences ☐ Ecological, evolutionary & environmental sciences

For a reference copy of the document with all sections, see nature.com/documents/nr-reporting-summary-flat.pdf

Life sciences study design

All studies must disclose on these points even when the disclosure is negative.

Sample size	The sample sizes were predetermined by power analysis using G*Power 3.1 (Faul, Erdfelder, Lang, & Buchner, 2007).
Data exclusions	Six participants were excluded from the fMRI analysis, including three who did not complete fMRI scanning and three who exhibited excessive head motion (i.e., over 3 mm in translation or 3 degrees in rotation). In the first tDCS experiment, eight were excluded either for requesting to abort the experiment or for having a mean RT that exceeded 2.5 SD from the group mean. In the second tDCS experiment, seven were excluded using the same criteria. In the TMS experiment, eight were excluded based on the same exclusion criteria used in the tDCS experiments.
Replication	All key behavioral, fMRI, and stimulation findings were successfully replicated across independent experiments, including a second HD-tDCS cohort with different stimuli; a neuronavigated TMS study further reproduced the causal effect with higher spatial specificity.
Randomization	Participants in this study were assigned to experimental groups using random allocation. This randomization ensured that group assignment was unbiased and that potential confounding variables were evenly distributed across groups.
Blinding	In the two tDCS experiments, blinding was implemented for both the stimulation and sham groups. After the experiment, participants completed a questionnaire assessing sensations such as itching, pain, and burning experienced during the tDCS session. Analysis revealed no significant differences in reported sensations between the stimulation and sham groups in both experiments, confirming successful blinding of participants to the stimulation conditions. In the TMS experiment, blinding was also implemented for both the stimulation and sham groups.

Reporting for specific materials, systems and methods

We require information from authors about some types of materials, experimental systems and methods used in many studies. Here, indicate whether each material, system or method listed is relevant to your study. If you are not sure if a list item applies to your research, read the appropriate section before selecting a response.

Materials & experimental systems

n/a	Involved in the study
☒	☐ Antibodies
☒	☐ Eukaryotic cell lines
☒	☐ Palaeontology and archaeology
☒	☐ Animals and other organisms
☒	☐ Clinical data
☒	☐ Dual use research of concern
☒	☐ Plants

Methods

n/a	Involved in the study
☒	☐ ChIP-seq
☒	☐ Flow cytometry
☐	☒ MRI-based neuroimaging

Plants

Seed stocks

Report on the source of all seed stocks or other plant material used. If applicable, state the seed stock centre and catalogue number. If plant specimens were collected from the field, describe the collection location, date and sampling procedures.

Novel plant genotypes

Describe the methods by which all novel plant genotypes were produced. This includes those generated by transgenic approaches, gene editing, chemical/radiation-based mutagenesis and hybridization. For transgenic lines, describe the transformation method, the number of independent lines analyzed and the generation upon which experiments were performed. For gene-edited lines, describe the editor used, the endogenous sequence targeted for editing, the targeting guide RNA sequence (if applicable) and how the editor was applied.

Authentication

Describe any authentication procedures for each seed stock used or novel genotype generated. Describe any experiments used to assess the effect of a mutation and, where applicable, how potential secondary effects (e.g. second site T-DNA insertions, mosiacism, off-target gene editing) were examined.

Magnetic resonance imaging

Experimental design

Design type

The attribute amnesia task was conducted using a slow event-related design. The localizer task was conducted using a block-designed.

Design specifications

The fMRI experiment consisted of two tasks: the attribute amnesia task and the localizer task. The attribute amnesia task included four runs, each with 32 trials and inter-trial intervals of 8-12 seconds. Each trial involved a fixation display (1 s), a stimulus presentation (1 s), masking (100 ms), and a variable fixation interval (8-12 s). Participants completed either regular trials, reporting the location of a target face, or surprise trials, identifying the target face before reporting its location. Feedback was provided after each response.The localizer task used a block design with four runs, each containing 10 randomized blocks (5 face blocks and 5 house blocks). Blocks were separated by 12-second rest periods. Each block consisted of 24 trials, with a stimulus presented for 400 ms followed by a 100 ms fixation cross.

Behavioral performance measures

We recorded participants' reaction times (RTs) and accuracy for two types of tasks: (1) location reporting in regular trials, where participants identified the target face's location by pressing one of four number keys (1-4), and (2) face identity selection in surprise trials, where participants identified the target face among four options and subsequently reported its location. These measures were used to evaluate task performance and ensure participant engagement.

Acquisition

Imaging type(s)

Functional and structural imaging.

Field strength

3 Tesla.

Sequence & imaging parameters

Functional imaging.: TR/TE/flip angle = 2,000 ms / 30 ms / 90°, FOV = 192 × 192 mm2, matrix size = 64 × 64, slice thickness = 3 mm, number of slices = 32. T1-weighted structural imaging: TR/TE/flip angle = 2,530 ms / 2.27 ms /7°, FOV = 256 × 256 mm2, matrix size = 256 × 256, slice thickness = 1 mm, number of slices=208.

Area of acquisition

Whole brain coverage.

Diffusion MRI ☐ Used ☒ Not used

Preprocessing

Preprocessing software	The AFNI software package (version 21.2.03, http://afni.nimh.nih.gov/afni) was employed for fMRI data analysis. The fMRI preprocessing steps included: slice timing correction; head motion correction using realignment on functional volumes; co-registration of the averaged structural image with functional images; non-linear transformation to the Montreal Neurological Institute (MNI) template; functional volumes were resampled into 3 × 3 × 3mm3 resolution; volumes with excessive motion were censored (marked as zeros, otherwise as ones) if the Euclidean norm of derivatives of motion parameters exceeded 0.3 mm; spatial smoothing was applied using a Gaussian filter with a 4 mm full-width half-maximum; and finally, the voxel time series were scaled to percent signal change.
Normalization	Normalized to MNI152 template using non-linear transformation.
Normalization template	MNI152 template.
Noise and artifact removal	Motion artifacts were addressed by censoring volumes with motion exceeding 0.3 mm (Euclidean norm of motion derivatives). Spatial smoothing with a 4 mm Gaussian filter was applied, and voxel time series were scaled to percent signal change to normalize baseline variability. Six motion regressors (three rotations and three translations) were included in the GLM.
Volume censoring	AFNI was used to censor volumes exceeding a motion threshold of 0.3 mm (Euclidean norm of derivatives).

Statistical modeling & inference

Model type and settings	A whole-brain analysis was conducted using a general linear model (GLM) implemented with the TENT function in AFNI's 3dDeconvolve. The GLM included 12 regressors: two experimental conditions (PRE and POST conditions) to estimate event-related BOLD signals, three additional task regressors, six motion regressors (three rotations and three translations), and a binarized censored time series to account for excessive motion.
Effect(s) tested	The analysis examined event-related BOLD signal differences between PRE and POST conditions. Voxel-wise paired t-tests were conducted for the contrast "PRE > POST," with significance at p < 0.01 (FDR corrected, cluster size > 40).

Specify type of analysis: ☐ Whole brain ☐ ROI-based ☒ Both

Anatomical location(s)	For the face-localizer task, a paired t-test map was obtained at the group level for the contrast "face > house" (threshold p < 0.01, FDR correction at the voxel level), identifying task-related activation. The anterior temporal lobe (ATL) was localized as the activated voxel cluster in the anterior part of the temporal lobe, and the fusiform face area (FFA) was identified as the activated voxel cluster in the middle fusiform gyrus, based on established criteria (Collins & Olson, 2014).
Statistic type for inference<br>(See Eklund et al. 2016)	Voxel-wise paired t-tests were conducted to assess condition contrasts at the group level, with statistical significance set at p < 0.01 (FDR corrected) at the voxel level. A minimum cluster size threshold of 40 voxels was applied for cluster-wise inference to ensure the reliability of activation patterns.
Correction	FDR corrected.

Models & analysis

n/a	Involved in the study
☐	☒ Functional and/or effective connectivity
☒	☐ Graph analysis
☒	☐ Multivariate modeling or predictive analysis

Functional and/or effective connectivity	Effective connectivity was analyzed using Dynamic Causal Modeling (DCM) in SPM12. Parameters C (driving input), A (intrinsic connections), and B (modulatory effects) were estimated to examine the connectivity between the SMG, ATL (or FFA), and dlPFC. Bayesian model comparison was performed to identify the most likely connectivity model, and modulatory effects were evaluated using one-sample t-tests. We also additionally conducted an extended data-driven analysis using Parametric Empirical Bayes (PEB) with Bayesian Model Averaging (BMA).
